# PReS13-SPK-1292: Gut microbiome and metabolism

**DOI:** 10.1186/1546-0096-11-S2-I32

**Published:** 2013-12-05

**Authors:** H Tilg

**Affiliations:** 1 Department of Internal Medicine I, Endocrinology, Gastroenterology and Metabolism, Medical University Innsbruck, Innsbruck, Austria

## 

The enormous number and diversity of microorganisms in the human gastrointestinal tract support the host in many functions such as digestion of complex carbohydrates. The relationship between the gut microbiota and energy homeostasis, metabolic dysfunction and inflammation and their role in the pathogenesis of obesity.-related disorders is increasingly recognized. Obesity developing in genetically or diet-induced obese mice is characterized by impressive changes in the composition and metabolic function of the gut microbiota. Importantly, colonization of germ-free mice with an "obese-gut-derived" microflora results in a much greater increase in total body fat and leads to obesity. Similar alterations as in experimental models have been observed in human obesity. The gut microbiota is able to directly regulate host gene expression and thereby could control host energy expenditure and storage. This may take place by various mechanisms such as regulation of Fiaf, AMPK or short chain fatty acids. Furthermore, it is increasingly recognized that diet may have a fundamental effect on the composition of our microbiota. The innate immune system is another important player controlling microbiota composition. Animals deficient for toll-like receptor 5 (TLR5) or certain members of inflammasomes such as Caspase1 or ASC develop a dysbiosis which is associated with obesity, metabolic syndrome and fatty liver disease. Whereas knowledge in various models is increasing, data in humans are still in its infancy. This is especially true for interventional studies manipulating the gut microbiota e.g. by using antibiotics, pro- or prebiotics. A first human study using fecal transplantation recently showed that insulin sensitivity might be improved via such a strategy. Overall, data suggest an important role for the microbiota in metabolic dysfunction, type 2 diabetes and obesity.

## Disclosure of interest

None declared.

